# Vasculogenic Mimicry Formation Predicts Tumor Progression in Oligodendroglioma

**DOI:** 10.3389/pore.2021.1609844

**Published:** 2021-08-18

**Authors:** Jing Xie, Xue Kong, Wei Wang, Yuan Li, Mengyu Lin, Heng Li, Jingjing Chen, Wenchao Zhou, Jie He, Haibo Wu

**Affiliations:** ^1^ School of Medicine, Shandong University, Jinan, China; ^2^ Department of Pathology, Anhui Provincial Hospital, Shandong University, Hefei, China; ^3^ Department of Pathology, the First Affiliated Hospital of USTC, Division of Life Sciences and Medicine, University of Science and Technology of China, Hefei, China; ^4^ Intelligent Pathology Institute, the First Affiliated Hospital of USTC, Division of Life Sciences and Medicine, University of Science and Technology of China, Hefei, China

**Keywords:** PFS, oligodendroglioma, vasculogenic mimicry, ATM, ATR

## Abstract

Vasculogenic mimicry (VM) has been identified as an important vasculogenic mechanism in malignant tumors, but little is known about its clinical meanings and mechanisms in oligodendroglioma. In this study, VM-positive cases were detected in 28 (20.6%) out of 136 oligodendroglioma samples, significantly associated with higher WHO grade, lower Karnofsky performance status (KPS) scores, and recurrent tumor (*p* < 0.001, *p* = 0.040, and *p* = 0.020 respectively). Patients with VM-positive oligodendroglioma had a shorter progress-free survival (PFS) compared with those with VM-negative tumor (*p* < 0.001), whereas no significant difference was detected in overall survival (OS) between these patients. High levels of phosphorylate serine/threonine kinases Ataxia-telangiectasia mutated (pATM) and phosphorylate Ataxia-telangiectasia and Rad3-Related (pATR) were detected in 31 (22.8%) and 34 (25.0%), respectively out of 136 oligodendroglioma samples. Higher expressions of pATM and pATR were both associated with a shorter PFS (*p* < 0.001 and *p* < 0.001). VM-positive oligodendroglioma specimens tended to exhibit higher pATM and pATR staining than VM-negative specimens (r_s_ = 0.435, *p* < 0.001 and r_s_ = 0.317, *p* < 0.001). Besides, Hypoxia-inducible factor-1α (HIF1α) expression was detected in 14(10.3%) samples, correlated with higher WHO grade and non-frontal lobe (*p* = 0.010 and *p* = 0.029). However, no obvious connection was detected between HIF1α expression and VM formation (*p* = 0.537). Finally, either univariate or multivariate analysis suggested that VM was an independent unfavorable predictor for oligodendroglioma patients (*p* < 0.001, HR = 7.928, 95%CI: 3.382–18.584, and *p* = 0.007, HR = 4.534, 95%CI: 1.504–13.675, respectively). VM is a potential prognosticator for tumor progression in oligodendroglioma patients. Phosphorylation of ATM and ATR linked to treatment-resistance may be associated with VM formation. The role of VM in tumor progression and the implication of pATM/pATR in VM formation may provide potential therapeutic targets for oligodendroglioma treatment.

## Introduction

Oligodendroglial tumors refer to diffuse infiltrating gliomas most commonly arising in the frontal lobe with a slight male predominance and a peak incidence aged 4th decade, which occupy the second place in intracranial glial tumors and are mainly composed of WHO grade Ⅱ and Ⅲ oligodendrogliomas (the latter usually named as anaplastic oligodendroglioma, AO) [1, 2]. The diagnosis of canonical oligodendroglioma requires demonstration of concurrent isocitrate dehydrogenase (IDH) one or two mutation and 1p/19q co-deletion, which predict not only better prognosis but also better response to chemotherapy [3, 4]. Despites the relatively favorable clinical prognosis of oligodendrogliomas, recurrence is inevitable and the outcome is ultimately fatal for virtually all patients [5]. Active vascular formation characterized by dense network of blood vessels in oligodendrogliomas indicates a potential relationship between angiogenesis and tumor progression. It has been reported that VEGF was unexpectedly expressed by tumor cells in addition to blood vessels in AOs, which contributes to tumor progression [6]. Overexpression of VEGF associated with active vascularization like endothelial hyperplasia was identified as a poor prognostic factor [7]. However, anti-angiogenesis therapy aiming at vascular endothelial cells (ECs) with the anti-VEGF receptor monoclonal antibody bevacizumab showed limited benefits in oligodendroglioma patients [8].

Recently a new form of tumor neovascularization termed vasculogenic mimicry (VM) has been identified as an important vasculogenic mechanism in aggressive tumors [9]. VM refers to a special and intricate meshwork composed of irregular micro-vascular channels absent of ECs lining and rich in extravascular depositions, anastomosing with host microcirculation to conduct blood flow [10]. VM formation shows a significant influence on tumor differentiation, invasion, and metastasis, so it presents as an unfavorable prognostic indicator in cancer patients [11, 12]. This alternative mechanism of angiogenesis utilized by tumor cells may be VEGF-independent and therefore could escape from antiangiogenic therapy targeting VEGF, which partially explains the disappointing results of traditional angiogenesis inhibitors [13–15]. Hence, in order to achieve a better therapeutic outcome in cancer patients, it is necessary to explore a more effective chemotherapy regimens targeting not only ECs but also VM. To date, VM has been identified in various aggressive tumors including AO, but the clinical meaning and the regulatory mechanisms in oligodendroglioma remain unknown [16].

Serine/threonine kinases Ataxia-telangiectasia mutated (ATM) and Ataxia-telangiectasia and Rad3-Related (ATR) are well known as major regulators in DNA damage response (DDR), both belonging to the phosphatidylinositol 3-kinase (PI3K)-related protein kinase (PIKK) family. They have cross-talks and take effect synergistically to activate downstream pathways [17]. Besides DDR, ATM and ATR involve in cellular homeostasis, immune regulation, senescence, tumorigenesis and tumor progression [18–22]. The activation of ATM and/or ATR in cancer cells contributes to treatment resistance and is evaluated as prognostic indexes [23–26]. Both ATM and ATR play important roles in pathological angiogenesis [27–28], but little is known about the involvement of ATM/ATR in VM formation.

In this study, we evaluated phosphorylate ATM (pATM) and phosphorylate ATR (pATR) levels using semi-quantitative immunohistochemical (IHC) staining and analyzed the VM formation by histochemical double-staining in a cohort of 136 surgically excised oligodendroglioma specimens. We further analyzed the relationship between these indexes and clinical parameters. Our results revealed the value of VM formation as a potential prognostic factor for tumor progression in oligodendroglioma patients. We also discovered a positive correlation between the presence of VM and the high pATM/pATR levels in oligodendrogliomas. We propose that pATM/pATR may contribute to treatment resistance through promoting VM formation.

## Materials and Methods

### Tumor Specimens

A total of 154 eligible surgically resected, formalin-fixed, paraffin-embedded (FFPE) oligodendroglioma specimens were collected from the Department of Pathology, the First Affiliated Hospital of USTC, Division of Life Sciences and Medicine, University of Science and Technology of China from 2010 to 2018. Diagnosis of oligodendroglioma was performed according to the 2016 revision of the WHO Classification of Tumors of CNS, with demonstration of IDH1 immune-positive and 1p/19q co-deletion concurrently. Clinical data of each case was summarized in Table 1. The follow-up period was from 12 to 110 months with a median time of 48 months. Progression-free survival (PFS) was defined as the time span from the date of surgery to the first relapse, secondary tumor occurrence, death, or last follow-up. Overall survival (OS) was evaluated from the date of surgery to death caused by tumor or last follow-up. 18 cases were excluded from the study because of incomplete clinical information. Finally, 136 PFS and OS data were collected in which 23 of patients had relapsed or secondary tumor and 18 died. This study was conducted with approval by the Ethics Committee of the First Affiliated Hospital of USTC and in accordance with the Declaration of Helsinki. The written informed consent was obtained from each patient.

**TABLE 1:** Association of VM, pATM and pATR expressions with clinical characteristics in oligodendroglioma patients.

Variable	VM			pATM			pATR		
	−	+	*p* value	Low	High	*p* value	Low	High	*p* value
Gender			0.745			0.957			0.842
Male	58	16		57	17		56	18	
Female	40	12		48	14		46	16	
Age (y, Median)	49	46	0.168	48	47	0.626	49	48	0.614
WHO			**<0.001**			**<0.001**			**0.002**
Ⅱ	75	7		72	10		69	13	
Ⅲ	33	21		33	21		33	21	
Reccurrent			**0.020**			**0.010**			**0.025**
Not	96	20		94	22		91	25	
Yes	12	8		11	9		11	9	
Side			0.638			0.776			0.872
left	41	12		39	14		39	14	
right	61	16		61	16		58	19	
middle	1	0		1	0		1	0	
bilateral	5	0		4	1		4	1	
Lobe			0.896			0.526			0.391
Frontal	68	17		64	21		67	18	
Temporal	7	2		6	3		6	3	
Parietal	2	1		3	0		3	0	
Lateral vertricle	2	0		2	0		1	1	
Corpus callosum	2	0		2	0		2	0	
Insular lobe	4	2		5	1		5	1	
Central region	4	0		4	0		3	1	
Intraspinal	1	0		1	0		1	0	
Multiple center	18	6		18	6		14	10	
KPS score			**0.040**			0.053			**0.002**
<70	71	24		69	26		64	31	
70–100	37	4		36	5		38	3	
TR			0.688			0.228			0.624
Yes	85	23		81	27		80	28	
No	23	5		24	4		22	6	
Relapse			**<0.001**			**<0.001**			**<0.001**
Absent	99	14		95	19		92	22	
Present	9	14		10	12		10	12	

Note: *p* < 0.05 was considered statistically significant and those values are shown in bold. Abbreviations: KPS, karnofsky performance status; pATM, phosphorylate serine/threonine kinases Ataxia-telangiectasia mutated; pATR, phosphorylate Ataxia-telangiectasia and Rad3-Related; TR, totally removed; VM, vasculogenic mimicry.

### IHC Staining and Scoring

Briefly, 3 µm thick slices of FFPE specimens were deparaffinized and hydrated through a series of xylenes and alcohols, followed by antigen retrieval and blocking. The slices were then incubated with primary antibodies including pATM (Abcam, ab81292, 1:1,600), pATR (Abcam, ab178407, 1:4,000) and Hypoxia-inducible factor-1α (HIF1α, ZSGB-BIO, ZA-0552) for 2 h at room temperature. Next, the slices were washed with PBS and incubated with avidin-biotin-peroxidase (MXB Biotechnologies, KIT-5220) at room temperature for 15 min followed by reaction with diaminobenzidine (MXB Biotechnologies, KIT-5220) for 1–2 min. Finally, all stained slices were washed with running tap water for 15 min and then counterstained with hematoxylin. pATM and pATR levels were analyzed in a semiquantitative manner *via* composite score system by assessing both the percentage and intensity of stained tumor cells. The percentage of positive cells was evaluated per high-power field (HPF) by ×400 magnification. The slices with 5% or less positive cells score 0 point. In the same way, each slice scores 1 (5–25%), 2 (26–50%), 3 (51–75%) or 4 (76–100%). In addition, stained signals in each slice score 0, 1, 2 or 3, corresponding to negative, weak, moderate or high intensity, respectively. Scores for the percentage and intensity were multiplied as the overall score of the staining. Tumor specimens were classified into low expression and high expression groups according to overall score: low expression indicates 0 to 3 points and high expression indicates 4 to 12 points. All slices were evaluated independently by two pathologists without knowledge of the identity and clinical outcome of patients.

### CD31/GFAP-Periodic Acid-Schiff Dual-Staining and VM Detection

Both CD31-PAS and CD34-PAS dual staining are widely and well accepted for detecting VM vessels. In our preliminary experiment, no significant difference was detected in vessles stained by these two method (data not shown). Considering the relatively restrictive expression of CD31 in endothelium and extensive spectrum expanding from immature to mature vascular compared with a occasionally nonspecific expression of CD34 in oligodendrogliomas, we chose CD31 as the major marker accompany with PAS for proving VM existence in this study. After IHC staining with the anti-CD31 monoclonal antibody (Cell Signaling Technology, #3528, 1:800), the slices were exposed to sodium periodate (BaSO, BA4080B) for 10 min, rinsed with distilled water for 5 min, and then incubated with Schiff solution (BaSO, BA4080B) for 5 min. Slices were counterstained with hematoxylin, dehydrated, and mounted. Stained sections were subjected to microscopy at a magnification of ×400 and analyzed by two pathologists without knowledge of demographic or outcome data. PAS-positive tubes lined with CD31-positive cells (PAS+/CD31+) were defined as endothelium-dependent vessels. VM was defined as PAS-positive tubes enclosed by tumor cells, with red blood cells (RBCs) in the lumens but without ECs lining. The absence of ECs in VM was confirmed by hematoxylin-eosin (H&E) staining and IHC staining with anti-CD31 antibodies (PAS+/CD31−). Each section was thoroughly checked to detect VM formation. VM ratio was counted which was expressed as a percentage of total vessels (VM vessels/total vessels per HPF). In addition, we performed GFAP/PAS dual-staining (anti-GFAP monoclonal antibody, ZSGB-BIO, ZA-0529), similar to the procedure mentioned above, to further confirm VM vessles which exhibited as PAS-positive micro-channels surrounded by tumor cells expressed GFAP strongly.

### Statistical Analysis

The ROC curve was utilized to determine the best cutoff value of VM ratio. The chi-square test or Fisher’s exact and *t*-test were used for categorical and continuous variables respectively. PFS and OS were evaluated by Kaplan-Meier analysis, and the difference between groups was determined by Log-rank test. *p* values less than 0.05 were considered statistically significant (*p < 0.05, **p < 0.01, ***p < 0.001). Spearman coefficient was applied to determine the correlation between pATM/pATR levels and VM formation. Cox proportional hazards regression model was applied to perform univariate and multivariate analyses. Variables with a significance of *p* < 0.05 in univariate analysis were included in the subsequent multivariate analysis using stepwise backward elimination procedures. All statistical analyses were performed using SPSS Statistics 22.0.

## Results

### VM Formation is Associated With Tumor Progression in Oligodendroglioma Patients

**FIGURE 1:**
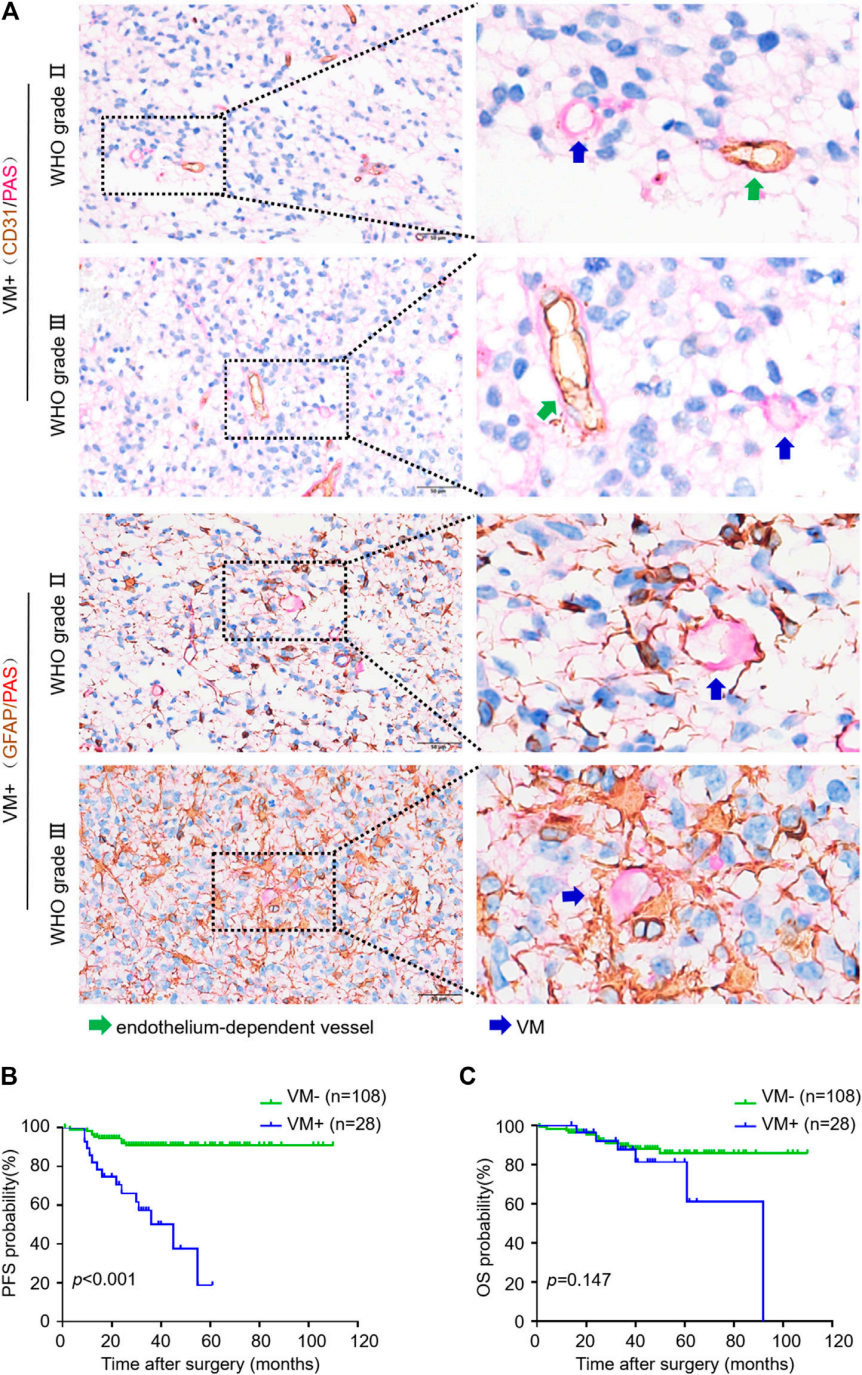
VM in oligodendroglioma negatively correlates with progression-free survival of patients. **(A)** CD31/PAS (above) and GFAP/PAS (below) dual-staining showing endothelium-dependent vessels and VM vessels in WHO Ⅱ and Ⅲ oligodendroglioma specimens, respectively. Green arrows indicate PAS+/CD31 + endothelium-dependent vessels. Blue arrows indicate PAS+/CD31- VM vessels (×400, scale bar: 50 μm). **(B,C)** Kaplan-Meier analysis of PFS **(B)** and OS **(C)** in oligodendroglioma patients with or without VM formation (*n* = 108 for VM−, n = 28 for VM+; *p* < 0.001 for PFS, *p* = 0.147 for OS).

We used CD31/PAS and GFAP/PAS dual-staining to distinguish CD31−/PAS+ and GFAP+/PAS + VM vessels from CD31+/PAS + endothelium-dependent vessels (Figure 1). Among the 136 oligodendroglioma specimens inspected, VM vessels was detected in 30 (22.1%) cases with 0–2 VM vessels/10HPF in each section. VM ratio ranged from 0 to 3.85% (mean 1.58%, media 1.43%). Over a range of VM ratios, receiver operating characteristic (ROC) analyze showed an area under the curve (AUC) of 0.743 for PFS and 0.582 for OS. A “cutoff” of 0.87% was yielded according to the greatest Youden index for PFS ROC (Supplementary Figure S1). Thus, we defined VM-positive as VM ratio greater than 0.87%. As a result, 28 cases were defined as VM-positive in this study. Chi-square test analysis suggested that VM-positive cases was significantly associated with higher WHO grade, lower Karnofsky performance status (KPS) score and recurrent tumor (*p* < 0.001, *p* = 0.040 and *p* = 0.020, respectively) (Table 1). In particular, 14 (50%) patients in the VM-positive group and 9 (8.3%) patients in the VM-negative group had tumor relapse (*p* < 0.001) (Table 1). Glomeruloid microvascular proliferation (GMP) that was commonly seen in high-grade gliomas was detected in 28 AOs. However, no obvious connection was identified between GMP and VM formation through Chi-square test (*p* = 0.199). Moreover, Kaplan-Meier analyses demonstrated that the VM-positive group had a shorter PFS relative to the VM-negative group (*p* < 0.001), although no significant difference was detected in OS between these two groups (Figure 1). These data indicate the potential value of VM formation in predicting prognosis of oligodendroglioma patients.

**FIGURE 2:**
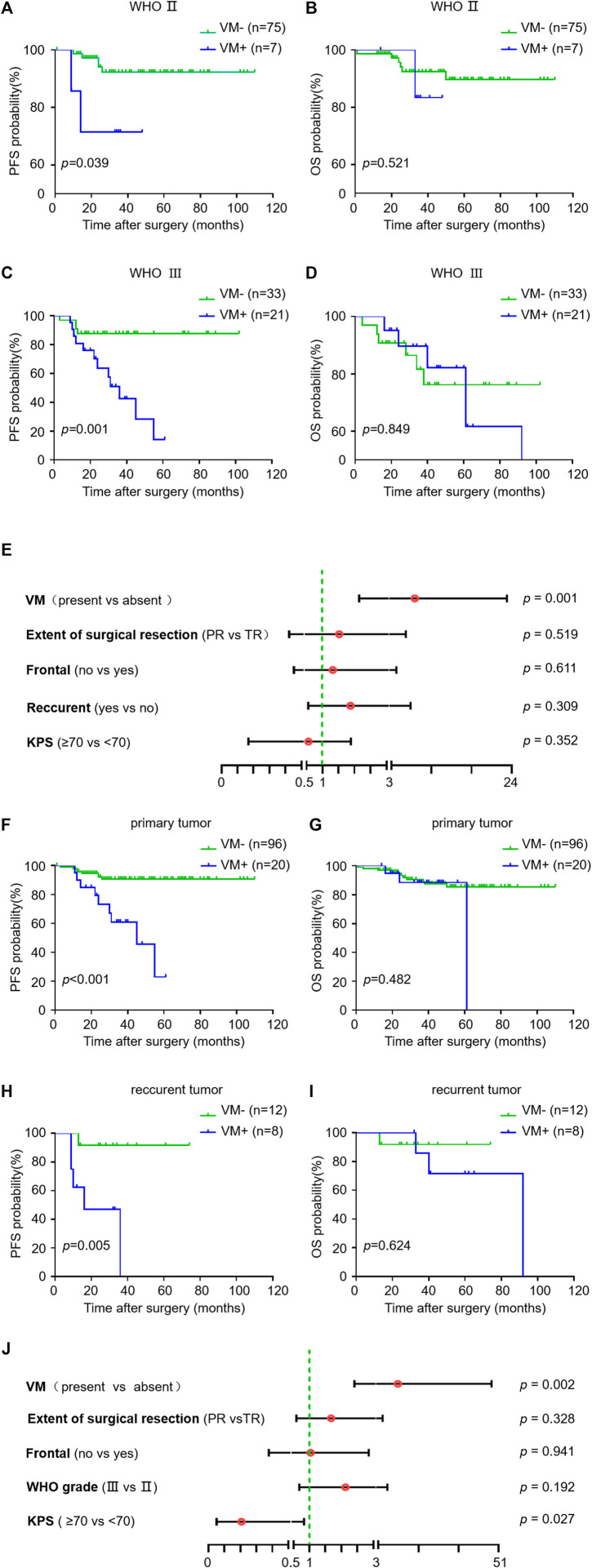
Relationship between VM and survival in subgroups of oligodendroglioma samples. **(A–I)** Kaplan-Meier analysis of PFS (left) and OS (right) in VM-positive and VM-negative patients with WHO Ⅱ oligodendroglioma **(A,B)** versus AO **(C,D)**, and primary **(F,G)** versus recurrent oligodendroglioma **(H,I)**. **(E,J)** Hazard ratios for PFS probabilities in AO patients **(E)** and primary oligodendroglioma patients **(J)**. *p* < 0.05 was considered as statistically significant.

We next investigated the impact of VM formation on prognosis in oligodendroglioma patients with different tumor grades as well as in primary and recurrent oligodendrogliomas. VM was correlated with PFS but not OS in WHO grade Ⅱ oligodendroglioma patients (*p* = 0.039 and *p* = 0.521). Likewise, in AOs, patients with VM formation had a significantly shorter PFS compared to those without VM formation (*p* = 0.001) (Figure 2). Stratified multivariate analyses were utilized to study the prognostic value of KPS, WHO grade, extent of surgical resection, tumor localization (frontal lobe or not), status of tumor (primary or recurrent) and VM formation [29]. The results showed an independent predictive impact of VM on PFS in AO patients (HR = 7.058, 95% CI: 2.148–23.189, *p* = 0.001). Meanwhile, VM formation was correlated with PFS but not OS both in primary and recurrent oligodendroglioma patients (*p <* 0.001 and *p* = 0.005) (Figure 2). Considering the potential bias due to the small number of recurrent oligodendroglioma patients in the statistical analysis, we performed stratified multivariate analyses only in primary oligodendroglioma patients. The results revealed an independent predictive impact of VM for PFS (HR = 10.778, 95% CI: 2.409–48.225, *p* = 0.002) (Figure 2). These results suggest that VM formation may affect the progression of oligodendroglioma but not the overall tumor growth, resulting in a shorter PFS but no impact on OS.

### Activation of ATM/ATR in Oligodendrogliomas Correlates With Tumor Grade and Recurrence

**FIGURE 3:**
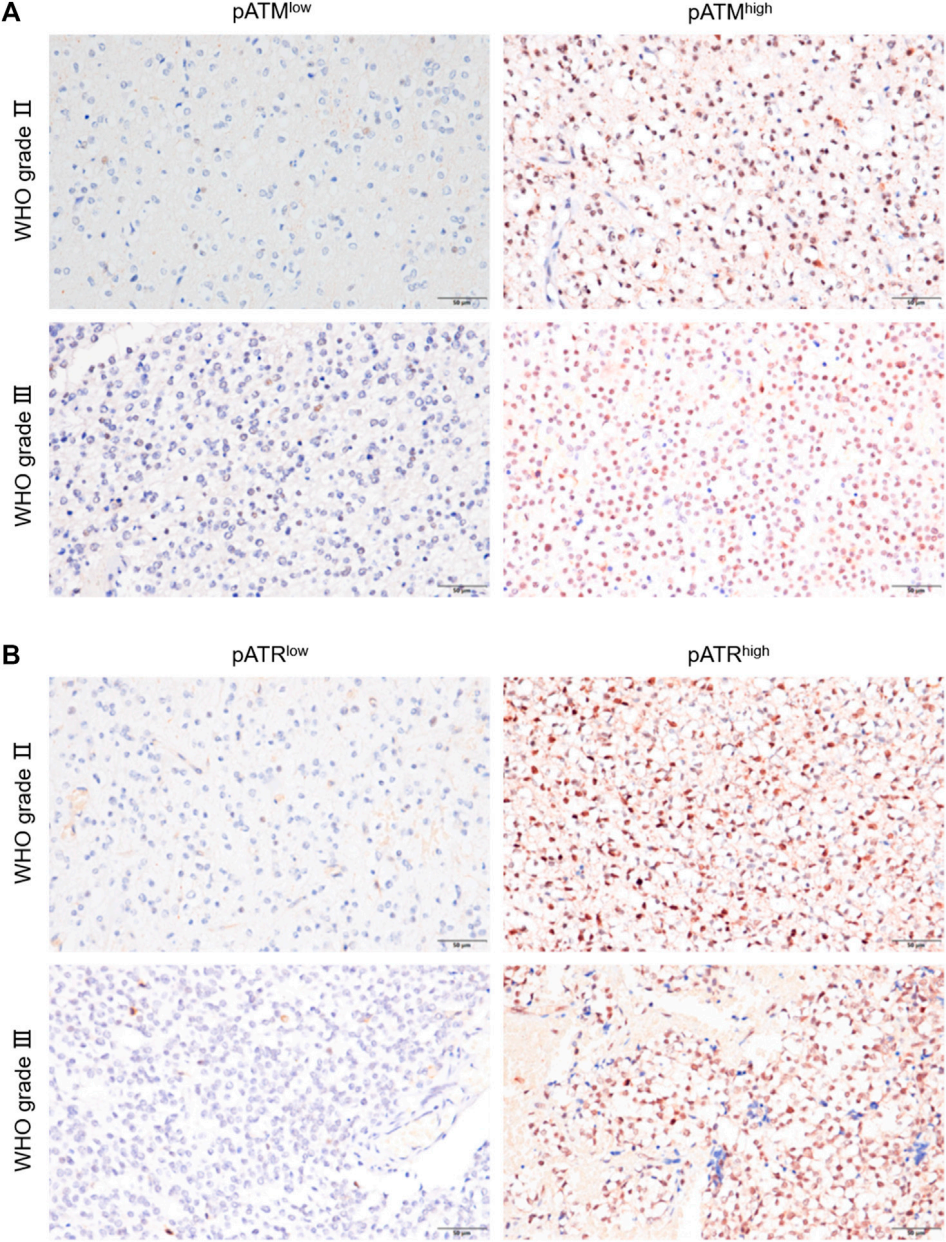
pATM and pATR expressions as well as their correlation with survival in oligodendroglioma samples. **(A,B)** Representative image showing IHC staining of pATM **(A)** and pATR **(B)** in WHOⅡ and WHOⅢ oligodendroglioma specimens respectively, the left for low and the right for high nuclear expression (×400, scale bar = 50 μm).

The activation of ATM and/or ATR contributes to treatment resistance and pathological neoangiogenesis in cancers. IHC of pATM and pATR was performed on oligodendroglioma specimens to determine the activation of ATM/ATR. pATM and pATR signals mainly localized in the nuclei of tumor cells (Figure 3). Low pATM was detected in 105 (77.2%), and high pATM was detected in 31 (22.8%) out of the 136 specimens. Meanwhile, low pATR was detected in 102 (75%), and high pATR was detected in 34 (25%) patients (Table 1). The correlation between the levels of pATM/pATR and clinicopathological features was investigated and summarized in Table 1. Higher pATM was significantly correlated with higher WHO grade (*p* < 0.001) and recurrent tumor (*p* = 0.01). pATR level demonstrated the same trend in terms of tumor grade (*p* = 0.002) and recurrence (*p* = 0.025), and higher level of it was associated with lower KPS scores (*p* = 0.002) (Table 1). No significant difference was detected between pATM/pATR levels and other clinical parameters including age, gender, predominant side or lobe of tumor location and the extent of surgical resection (Table 1). The results remind us the correlation between VM formation and tumor grade and recurrence, suggesting a probable link between VM formation and ATM/ATR activation.

### Activation of ATM/ATR But Not HIF1α Correlates With VM Formation in Oligodendrogliomas

**FIGURE 4:**
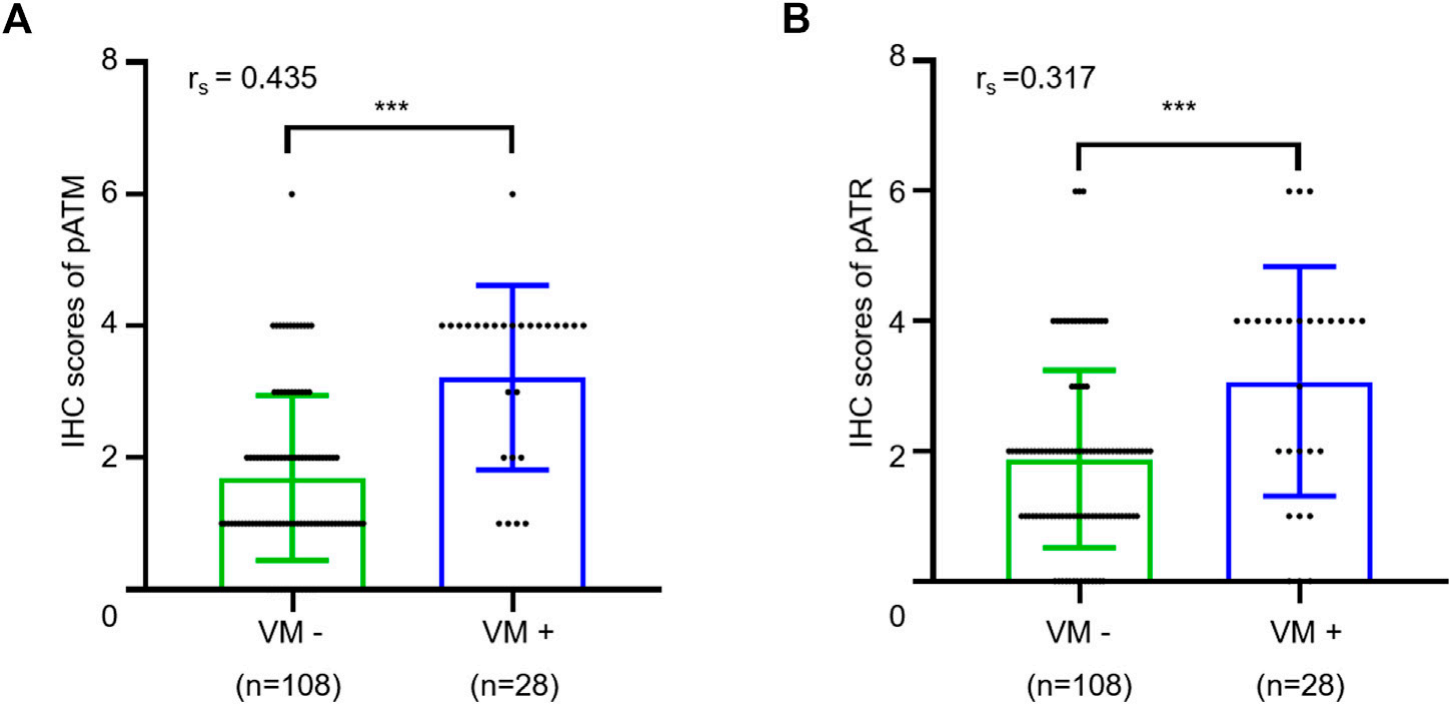
Relationship between VM and expressions of pATM and pATR. **(A,B)** The scatter plot combined with histogram showed that VM-positive cases had higher expression levels of pATM **(A)** or pATR **(B)** than VM-negative ones (*p* < 0.001 and *p* < 0.001). Quantum analysis of correlation coefficient showed a positive linear correlation exists between VM and expressions of pATM or pATR (*p* < 0.001, r_s_ = 0.435 and *p* < 0.001, r_s_ = 0.317).

Next, we sought to explore the potential involvement of ATM/ATR in VM formation in oligodendrogliomas. We determined the IHC scores in oligodendroglioma sections with or without VM formation. The results showed that VM-positive specimens exhibited higher pATM and pATR scores relative to VM-negative specimens (*p* < 0.001 for pATM and *p* < 0.001 for pATR). Moreover, there was a positive correlation between pATM/pATR levels and VM formation according to Spearman correlation analysis (r_s_ = 0.435, *p* < 0.001 and r_s_ = 0.317, *p* < 0.001) (Figure 4). As the classical downstream effector molecule in the ATM/ATR signaling pathway, HIF1α was examined by immumohistochemical staining as well. The results showed that only 14 cases expressed HIF1α with weak-moderate staining (Supplementary Figure S2). HIF1α expression was positively correlated with higher WHO grade and non-frontal lobe (*p =* 0.010, *p =* 0.029), but not correlated with VM formation (*p =* 0.537). In summary, activation of ATM/ATR but not HIF1α shows a positive correlation with VM formation in oligodendroglioma patients.

### Activation of ATM/ATR is Associated With Tumor Progression in Oligodendroglioma Patients

**FIGURE 5:**
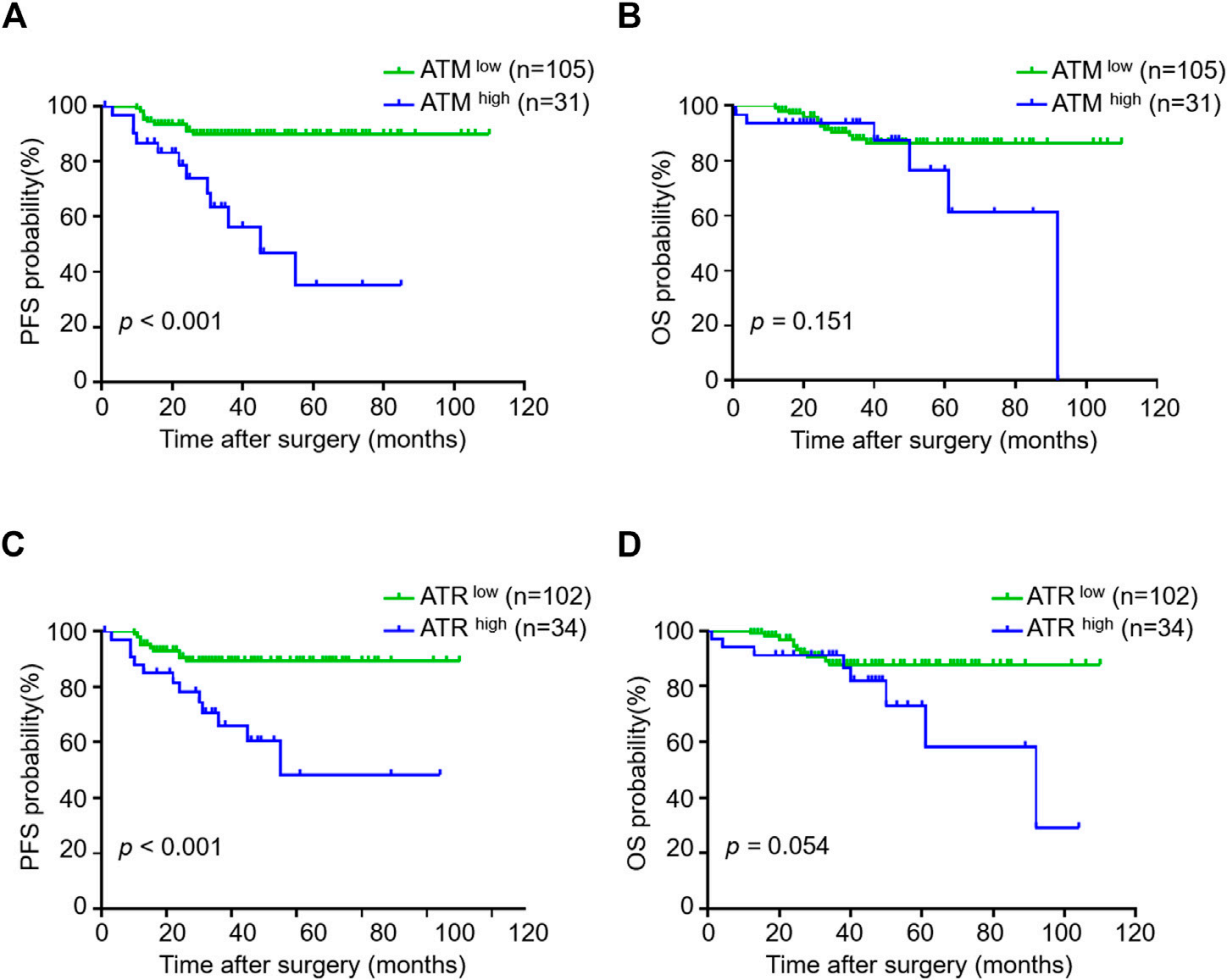
The correlation between pATM/pATR expressions and survival in oligodendroglioma samples. **(A–D)** Kaplan-Meier analysis of PFS (left) and OS (right) in oligodendroglioma patients grouping by pATM **(A,B)** and pATR **(C,D)** expressions.

Because VM formation is associated with tumor progression and pATM/pATR correlates with VM formation, we investigated the association between pATM/pATR and tumor progression in oligodendroglioma patients. Oligodendrogliomas with high pATM/pATR had an obvious tendency to relapse compared to those with low pATM/pATR (*p* < 0.001 for pATM and *p* < 0.001 for pATR) (Table 1). Patients with high pATM/pATR levels had a shorter PFS relative to those with low pATM/pATR levels (*p* < 0.001 for pATM and *p* < 0.001 for pATR), but no significant difference in OS was observed (*p* = 0.151 for pATM and *p* = 0.054 for pATR) (Figure 5). In patients who received chemotherapy or radiation-therapy, high pATM/pATR levels were also associated with shorter PFS (Supplementary Figure S3). Taken together, the levels of pATM/pATR in oligodendrogliomas may be associated with the progression but not overall growth of tumors, which recapitulates the situation of VM formation.

### VM Formation and pATM/pATR Predict Poor Outcomes

We then assess the prognostic and predictive values of VM, pATM/pATR and other indicators by using Cox proportional hazards regression model. Univariate analysis showed that higher WHO grade (*p* = 0.002), recurrent tumor (*p* = 0.040), high pATM (*p* < 0.001), high pATR (*p* = 0.002) and VM formation (*p* < 0.001) were unfavorable prognostic factors for PFS in oligodendroglioma patients. Multivariate analysis containing these five factors revealed that VM formation is an independent unfavorable predictor for tumor progression in oligodendroglioma patients (*p* = 0.007, HR = 4.534, 95% CI: 1.504–13.675) (Table 2).

**TABLE 2:** Univariate and multivariate Cox analysis of predictive factors for PFS in oligodendroglioma patients.

Variable	Univariate analysis			Multivariate analysis		
	HR	95% CI	*p* value	HR	95% CI	*p* value
VM			**<0.001**			**0.007**
negative	1	—	—	1	—	
positive	7.928	3.382–18.584		4.534	1.504–13.675	
WHO			**0.002**			0.222
Ⅱ	1	—		1	—	
Ⅲ	4.168	1.170–10.159		1.876	0.684–5.145	
Reccurrence			**0.040**			0.285
No	1	—		1	—	
Yes	2.668	1.046–6.810		1.695	0.645–4.456	
pATM			**<0.001**			0.727
low	1	—		1	—	
high	4.960	2.175–11.312		1.236	0.377–4.046	
pATR			**0.002**			0.682
low	1	—		1	—	
high	3.676	1.618–8.354		1.251	0.429–3.655	

Note: *p* < 0.05 was considered statistically significant and those values are shown in bold. Abbreviations: CI, confidence intervals; HR, hazard-ratio; pATM, phosphorylate serine/threonine kinases ataxia-telangiectasia mutated; pATR, phosphorylate ataxia-telangiectasia and Rad3-Related; VM, vasculogenic mimicry.

## Discussion

The concept of VM was brought forward to describe a phenomenon that tumor cells with a multipotent and stem cell-like phenotype form extracellular matrix (ECM)-rich and patterned vasculogenic-like networks by themselves, mimicking endothelial cells, to give an alternative perfusion way for aggressive tumors. This unique *de novo* vasculature formation process compensates for the lack of access to nutrient in avascular tumors and often promotes cancer invasion and metastasis [30]. Previous reports have suggested a correlation between VM formation and poorer prognosis of patients with breast, bladder, gastric and brain cancers [31–34]. In this study, we demonstrated that VM formation was associated with PFS of oligodendroglioma patients. In other words, patients with VM formation were more likely to suffer from tumor relapse, suggesting that VM may promote tumor progression. It has been reported that chemotherapeutic stress can promote VM, which in turn mediates resistance to anti-angiogenic therapy [35, 36]. VM formation has been blamed for the unexpected adverse outcome after anti-angiogenic treatment in the triple-negative breast cancer, uveal melanoma and ovarian cancer [37–39]. Our study suggests that VM formation should be taken into account when applying adjuvant therapy in oligodendroglioma patients.

ATM and ATR signaling is deeply involved in multiple processes in tumor biology [40, 41]. We found that high levels of pATM/pATR preferentially occurred in oliogodendrogliomas with VM formation. In addition, pATM/pATR levels correlated to a shorter PFS, which recapitulate the situation of VM formation. Importantly, pATM/pATR and VM were positively associated with tumor recurrence. ATM and ATR were reported play vital roles in treatment-resistance in gliomas [42–44]. pATM/pATR may promote resistance against chemoradiotherapy probably through enhancing DNA damage repair, leading to a poorer therapeutic outcome in oligodendroglioma patients. Besides, oligodendrogliomas with VM formation displayed higher pATM/pATR levels, suggesting the extra participation of ATM/ATR activation in VM formation. VM formation is regulated by intricate factors including hypoxia, epithelial-mesenchymal transition (EMT), angiogenic signaling, cancer stemness, matrix metalloproteinase substances, cell-adhesion and autophagy [45, 46]. ATM/ATR may be involved in multiple processes upstream of VM formation. ATM regulates autophagy in response to ROS perturbations [47]. Likewise, ATR is required for DNA damage-induced autophagy that utilizes lysosomes to degrade damaged organelles and macromolecules to facilitate VM formation [48]. Moreover, ATM supports the maintenance of cancer stem cells (CSCs) with the capacity to convert into EC-like cells to form VM, which in turn provides a niche to protect and nourish CSCs [49, 50]. Furthermore, it has been published that activated ATM/ATR helps cellular adaptation to hypoxia through phosphorylating or upregulating HIF-1 that promotes VM formation by activating transcription of a series of relevant genes [51, 52]. However, in oligodendroglioma specimens, maybe due to the relatively lower progression or richer vascular network, activation of HIF1α was too weak and its poor expression did not associate with VM formation. In short, VM formation during the progression of oligodendrogliomas may be regulated by pATM/pATR that participates in multiple essential processes upstream of VM independent of HIF1α.

In summary, our study revealed VM as a potential diagnostic index for tumor progression and relapse in oligodendrogliomas. The positive correlation between pATM/pATR and VM formation, as well as their similar value in predicting oligodendroglioma progression and recurrence, strongly suggests that pATM/pATR may contribute to VM formation. pATM/pATR may be an attractive therapeutic target to inhibit VM formation in postoperative treatment of oligodendroglioma patients.

## Conclusion

VM presents as a potential unfavorable predictor for progression in oligodendroglioma patients. Activated ATM and ATR are supposed to participate in VM formation which is implied by the positive correlation between them. The possible involvement of ATM and ATR in VM formation offers new insights into the therapeutic targets for oligodendroglioma patients especially for refractory and drug-resistance ones.

## Data Availability

The original contributions presented in the study are included in the article/Supplementary Material, further inquiries can be directed to the corresponding authors.

## Supplementary Material

Supplementary Figure 1 Receiver operating characteristic (ROC) analysis for PFS (left) and OS (right) of VM ratio. (**A,B**) Area under the curve (AUC) of PFS **(A)** and OS **(B)** showing 0.743 and 0.582, respectively.

Supplementary Figure 2 HIF1α expressions in oligodendroglioma samples. **(A)** Representative images showing IHC staining of HIF1α in WHOⅡ(above) and Ⅲ(below) oligodendroglioma specimens respectively, the left for negative and the right for positive nuclear expression (×400, scale bar = 50 μm).

Supplementary Figure 3 The correlation between pATM/pATR expressions and PFS in subgroups of oligodendroglioma samples. **(A–D)** Kaplan–Meier analysis of PFS in patients who received chemotherapy (above) or radiotherapy (below) grouping by different expressions of pATM **(A,C)** and pATR **(B,D)**.
