# Supplementary figures and images for: Vasculogenic Mimicry Formation Predicts Tumor Progression in Oligodendroglioma
Source: Pathol Oncol Res. 2021 Aug 18;27:1609844. doi: 10.3389/pore.2021.1609844 (PMC8408314; doi:10.3389/pore.2021.1609844)

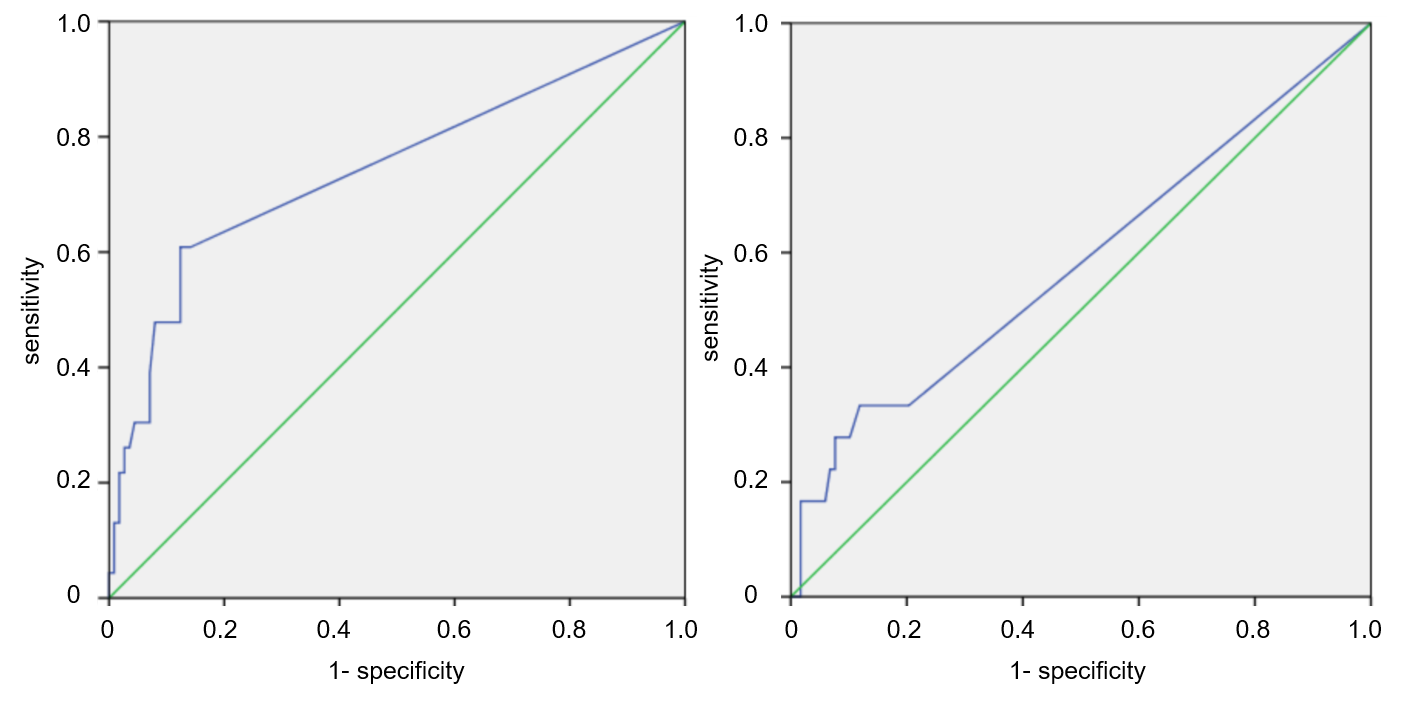

Supplement: Supplementary file 1 [file Image1.TIF]

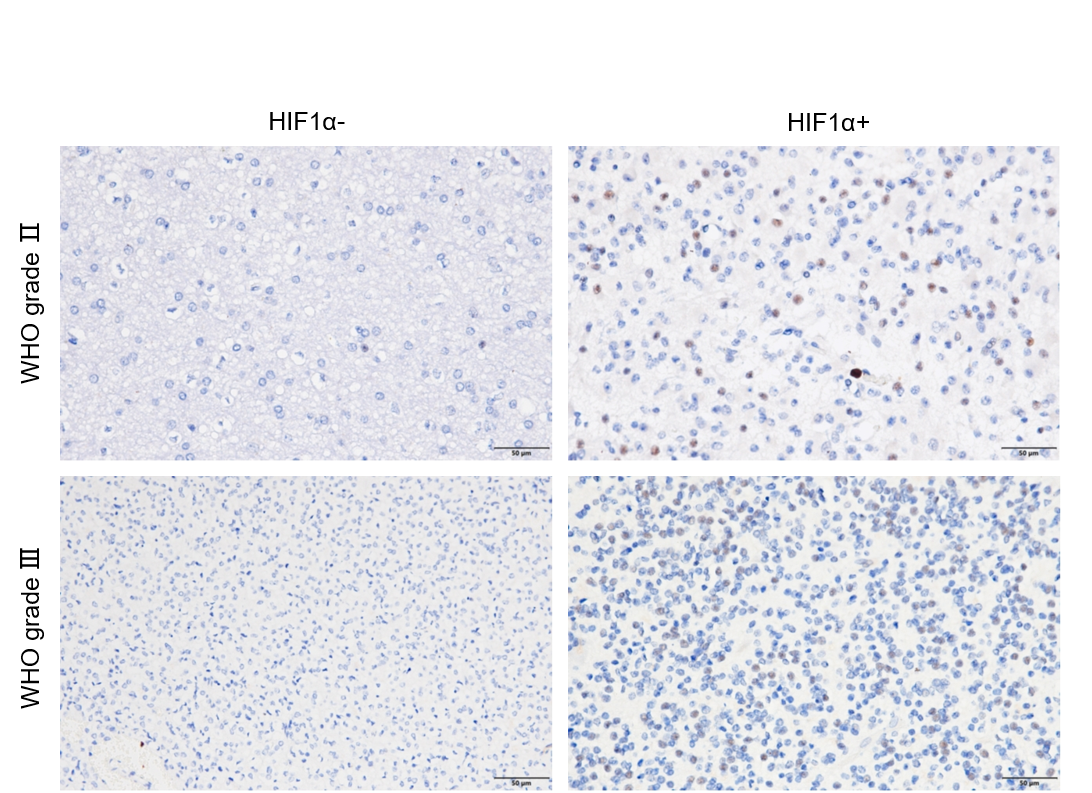

Supplement: Supplementary file 2 [file Image2.TIF]

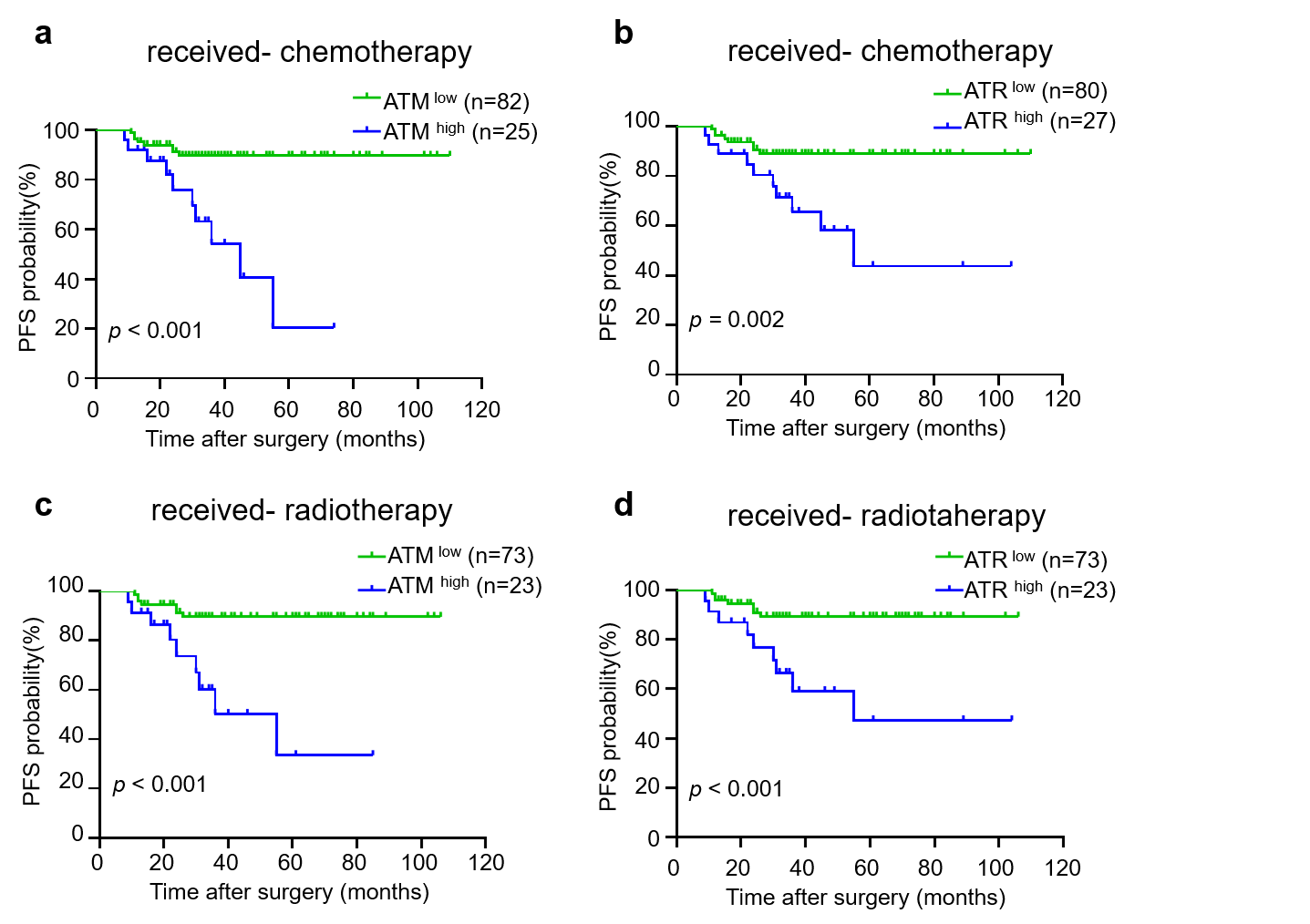

Supplement: Supplementary file 3 [file Image3.TIF]
